# “It’s why I’m the doctor that I am”: a qualitative study of the long-term outcomes of a longitudinal service-learning medical education program

**DOI:** 10.1186/s12909-026-10117-w

**Published:** 2026-08-10

**Authors:** Nichole M Waltrich, Joanna L Michel, Emma Rudd, Mayra Miranda, Izziah Thabath, Nicole A Perez, Monica Vela

**Affiliations:** 1 Urban Medicine Program, University of Illinois College of Medicine, Chicago, IL USA; 2 Department of Medical Education, University of Illinois College of Medicine, Chicago, IL USA; 3 Hispanic Center of Excellence, University of Illinois College of Medicine, Chicago, IL USA

**Keywords:** Undergraduate medical education, Service-learning, Health equity, Community-engaged learning, Structural competency, Professional identity formation, Qualitative research

## Abstract

**Background:**

Service-learning (SL) is widely used in medical education to promote structural competency and engagement with underserved communities. However, most SL evaluations focus on short-term outcomes, leaving limited understanding of whether these experiences shape physicians’ perspectives and practice over time. This study examined the long-term impact of the Urban Medicine (UMed) Program, a longitudinal service-learning program, on graduates’ professional development and commitment to health equity.

**Methods:**

A retrospective qualitative study was conducted using semi-structured interviews with 35 UMed alumni who graduated from the University of Illinois College of Medicine between 2009 and 2020 and had at least four years of postgraduate clinical experience. Interviews were conducted via Zoom. Data were analyzed inductively using thematic coding, with codes and themes refined through iterative discussion among the research team.

**Results:**

Graduates described UMed as having a sustained influence on their professional identity, clinical practice, and commitment to underserved communities. Three major themes emerged: (1) sustained community engagement through the longitudinal community rotation reinforced graduates’ sense of purpose and accountability to underserved populations; (2) structural competency training shaped participants’ approaches to patient care by increasing attention to social determinants of health, advocacy, and systemic barriers to care across specialties; and (3) cohort relationships and mentorship fostered belonging and provided ongoing professional support that helped sustain equity-oriented commitments throughout training and practice. Participants also described continued collaborations with peers and community partners that influenced career decisions and professional trajectories.

**Conclusions:**

Longitudinal service-learning programs may have durable effects on physicians’ professional development and clinical practice. Sustained community engagement, structural competency training, mentorship, and cohort-based learning environments may help reinforce commitments to health equity and support physicians in addressing systemic barriers to care across diverse clinical settings.

**Supplementary Information:**

The online version contains supplementary material available at https://doi.org/10.1186/s12909-026-10117-w.

## Introduction

### Background

Health inequities remain a persistent global challenge, prompting medical schools to adopt educational approaches that prepare future physicians to address the social and structural factors shaping patient health [1]. Service-learning (SL) has emerged as a prominent strategy, integrating meaningful community service with explicit learning objectives and reflection, while emphasizing reciprocal partnerships between institutions and communities [2]. Structural competency, the ability to recognize how social, economic, and political forces shape clinical presentations, has become an important guiding framework for understanding how such educational experiences prepare students to address health inequities [3]. Furthermore, longitudinal SL experiences may be particularly effective in supporting the application of structural competency by connecting classroom learning to sustained community engagement [4]. 

SL has been associated with improved understanding of population health, empathy, compassion, altruism, and commitment to underserved communities [5, 6]. Through direct engagement with communities, it can complement traditional didactic curricula and address concerns that medical training can diminish empathy, increase cynicism, and narrow focus to biomedical priorities at the expense of social considerations [5, 7–14]. Without intentional reinforcement, early commitments to health equity may not persist.

Emerging research suggests that reflective, longitudinal, community-engaged learning may mitigate these declines in commitment to health equity [14–20]. However, most evaluations of SL programs focus on short-term outcomes during medical school, limiting understanding of how SL influences physicians’ perspectives and practice over time [3, 14, 21]. In particular, it remains unclear whether longitudinal SL experiences sustain equity-oriented values beyond graduation. Understanding these long-term effects is critical for designing curricula that promote health equity and translate into durable changes in professional practice.

This study addressed the research question, “What themes emerge as reinforcing concepts for health equity training from medical students who graduate from a service-learning program? ?” The objective was to examine UMed’s impact on graduates’ educational experiences and how it influenced their subsequent practice and leadership decisions. To address this objective, we conducted a retrospective qualitative study using semi-structured interviews with program alumni.

### Program description

The Urban Medicine (UMed) Program at the University of Illinois College of Medicine (UICOM) in Chicago was established in 2005 to address early calls for health equity in medical education. Based on Kolb’s Experiential Learning Theory, UMed combines public health curricula with experiential community-based learning [22]. Its curricular themes are healthcare disparities, community engagement, diversity and intercultural communication, and policy and advocacy, taught and integrated over all 4 years of medical school [23]. Structural competency is introduced in the classroom and reinforced through the Longitudinal Community Rotation (LCR), the service-learning component of UMed. As part of the LCR, student teams partner with UMed’s longstanding community partnerships over all four years of medical school. Cohorts are guided by faculty and site mentors, including physicians, community members, and health service coordinators. Participation in UMed is voluntary, though it has a competitive application process admitting only 18 students per year. Prior evaluations of UMed suggest that longitudinal, experiential SL prepares students for leadership in advocacy, research, and policy [20]. This study builds on that work by examining how graduates apply these competencies in their subsequent professional practice.

## Methods

### Approach

This qualitative, retrospective study explored the long-term impact of the UMed Program on graduates’ professional development and clinical practice. A semi-structured interview approach was chosen for its ability to balance flexibility with consistency across interviews while allowing for a deep exploration of participants’ perspectives and discovery of unexpected themes [24]. The research team consisted of three faculty members, one UMed staff member, and three UMed Graduate Research Assistants (GRAs). GRAs conducted interviews, as they had no prior contact with participants, thereby mitigating potential bias. Two faculty members unaffiliated with UMed were involved in the interview guide creation and data analysis to enhance rigor and objectivity. The semi-structured interview guide (Appendix A in Supplementary Material) was adapted from Hand, et al. to account for UMed’s unique programmatic features [21]. While the guide was not formally piloted, the research team met in the early stages of interviewing to refine questions for grammar and language clarity. No substantive content was changed. This qualitative approach was justified because the research question required in-depth exploration of graduates’ experiences and the long-term influence of UMed on their practice and leadership decisions.

#### Reflexivity

The research team reflected on their positionality throughout the study. Faculty and staff members involved in UMed brought important programmatic knowledge but also recognized the potential for this proximity to shape interpretation, whereas GRAs and non-affiliated faculty members brought a more etic perspective to the research and analysis. Team discussions were held regularly to identify potential biases, ensure consistency in questioning, and critically examine interpretations during coding and theme development.

### Sampling and recruitment

Between 2009 and 2024, 328 students completed the UMed program. Inclusion criteria required graduates to have ≥ 4 years post-graduate clinical experience, resulting in 256 eligible participants. Recruitment occurred via email, with follow-ups via LinkedIn when email addresses were outdated. A total of 35 graduates participated, representing the demographics and specialties of the broader eligible cohort. Participants’ demographics (race, ethnicity, and gender) were collected from UMed Program records, and participants received a $50 gift card for participation.

### Data collection

Interviews were conducted by GRAs via Zoom and lasted approximately 30–60 min. Transcripts were auto-generated via Zoom and verified by research team members. All transcripts were de-identified before being uploaded to Dedoose (version 9.2.12) for analysis. Data saturation was reached when responses became redundant, no new themes emerged, and the study sample was representative of overall graduate demographics and specialties (Table 1). As part of recruitment, participants were asked to report on their current title and practice location.

**Table 1:** Demographic and specialty characteristics of Urban Medicine (UMed) Program graduate study participants, 2009–2020 (*n* = 35)

	No.	%
Gender		
Female	20	57%
Male	15	43%
Race/Ethnicity		
White	12	34.29%
Hispanic/Latino	8	22.86%
Black/African American	5	14.29%
Asian	8	22.86%
American Indian/Alaska Native	1	2.86%
Multiple R/E	0	0.00%
Unreported	1	2.86%
Years since graduating		
4 years post-grad	6	17.14%
5–9 years post-grad	12	34.29%
10 + years post-grad	17	51.43%
Specialty		
Internal Medicine	7	20.00%
Family Medicine	7	20.00%
OBGYN	5	14.29%
Pediatrics	4	11.43%
Emergency Medicine	3	8.57%
Psychiatry	3	8.57%
Surgery	3	8.57%
IM-Peds	1	2.86%
Neurology	1	2.86%
Orthopedic Surgery	1	2.86%
Residency and/or current practice locale		
Urban	33	94.28%
Suburban	1	2.86%
Rural	1	2.86%

### Data analysis

Using an inductive approach, four authors independently analyzed the same seven transcripts to identify preliminary themes and establish an initial codebook. Once consensus was reached on the coding strategy and terminology, these procedures were systematically documented to ensure consistency. One GRA coded the remaining 28 transcripts. Regular team meetings were held to refine themes, resolve discrepancies, and maintain an audit trail of decisions. Pseudonyms were assigned to participants, and quotes were lightly edited for clarity.

### Ethics

The University of Illinois Chicago Institutional Review Board approved this study (Protocol #2014 − 0981). Participants provided informed consent, and confidentiality was maintained through de-identification of transcripts and secure storage of data. All procedures adhered to ethical principles for human subjects research.

## Results

Data from 35 UMed graduates suggests that the program’s influence on professional development and clinical practice operates through direct and indirect pathways. Participants described UMed as paramount to their development as a physician as well as to sustaining their commitments to equity and community engagement within a broader medical training environment that often did not reinforce these values. For many participants, the LCR functioned as a critical counterbalance to the predominantly biomedical focus of medical training. Three major themes emerged concerning this developmental process and graduates’ long-term outcomes: (1) sustaining commitment to community engagement through the LCR, (2) integrating structural competency in clinical practice, and (3) fostering belonging and support through cohort relationships and faculty mentorship.

### Sustaining commitment to community engagement through the LCR

Graduates described their LCR as rare opportunities to gain real-world experience addressing health disparities and collaborating with local community organizations during medical school. The LCR allowed some graduates to reflect on their ideal roles as medical professionals in urban communities while transforming these visions into achievable realities. Greg, a psychiatrist (Class of (CO) 2020), claimed that his LCR “helped with the grounding and keeping the big picture in mind about medicine (…) it really helps recenter what medicine’s all about for me.” Janet, who practices family medicine (CO 2013), appreciated how her LCR affirmed her professional goals:

I think being in UMed was part of what gave me the space to really pursue my work with [partner organization] and also gave me the space to explore what kind of a career in medicine would be fulfilling for me, would make me excited to get up in the morning, would give me something that I’m really passionate about that could make a substantive difference in people’s lives in the community around me. So I think that experience that I got through the [LCR] was invaluable and has definitely influenced where, for me, my trajectory has gone.

For those graduates who entered UMed with a particular penchant for service, LCRs provided a necessary outlet for nurturing their predispositions. Erika, a pediatrician (CO 2020), characterized her LCR as “nurturing and feeding this other part of me that really wanted to be authentically involved in the community I was working in that I know I wouldn’t have gotten if I was just doing the med school part.” These longitudinal experiences allowed graduates to connect their academic training with their professional and personal values throughout their medical education, reinforcing their sense of purpose in medicine.

Ten research participants (28%) reported being from a suburban or rural hometown. For graduates with less experience working with urban and underserved communities or less familiarity with specific health disparities, exposure to local Chicago communities through their LCRs was described as both enlightening and pivotal to their careers. Ken (CO 2013), an emergency medicine physician, shared that his LCR, which involved delivering diabetes education programming, was “an opportunity for me to learn about the extent of healthcare inequity (…). It helped me as an Asian American provider, as a person, to reflect upon the implicit bias, even among care provided to Asian Americans.” Andrew (CO 2012), a family medicine physician, commented that his LCR “has really informed and colored and probably given me information that I wouldn’t otherwise have about what that life is like for those folks.” These experiences revealed the profound impact of first-hand exposure to working with underserved communities, helping graduates better understand patients’ challenges and how to approach care with greater empathy and awareness. Prominently, 94% of research participants were either completing residency or practicing full-time in urban settings.

A widely shared belief among interviewed graduates was that longitudinal community relationships forged through their LCR fostered a sense of accountability to uphold UMed tenets in their current practice. For some, this sense of accountability inspired ongoing community engagement. Britney (CO 2012), a family medicine physician, shared that the LCR operated partly as an “exposure to the community to remind and help, to either teach or reinforce, upcoming physician leaders that physicians don’t have all the answers, we learn a lot from the community.” Megan (CO 2016), an OB-GYN who worked with an organization that provided support to refugees, asylum seekers, and immigrants as part of her LCR, remarked that it was the “direct communication, doing things on the grassroots level with them that really led me to practice cultural awareness and to have a greater understanding of it.” Finally, Martha (CO 2015), a neurologist, claimed that her LCR project “instilled in me this desire to keep on doing these kinds of things, specifically community outreach,” a sentiment that underscores the long-term impact of LCRs on graduates’ professional trajectories.

### Integrating structural competency into clinical practice

Graduates emphasized that their UMed experiences shaped their approach to patient care, enabling them to consider the social determinants of health (SDOH) beyond traditional clinical parameters, regardless of specialty. Simona, an orthopedic surgeon (CO 2012), shared the following:

Patients who come to us for elective surgery are oftentimes struggling on multiple levels– financially, socially, health literacy, their basic access to primary care may be very limited. (…) I think my background with UMed and the associated experiences gave me the confidence to say, you know what, although it would be really easy to recommend elective surgery for this patient, let’s take a step back and be sure that this patient has the appropriate social resources, that they have access to primary care, so they can be in an optimized state of health prior to undergoing this elective surgery, and by doing so I feel like it allows me to provide higher quality care to this population.

Furthermore, some graduates highlighted their expanded view of physicians’ roles beyond the four walls of a clinic. For example, Mark (CO 2016), a surgeon, described advocating for vulnerable workers:

If you’re an occupational health physician, and you see that vulnerable populations are being manipulated into bad jobs that don’t pay well and are dangerous, then it’s our responsibility. (…) It’s the responsibility of the physician to actually go to the companies, to the temp agencies involved and ensure that the vulnerable population is protected from exploitation. So, you go find out what the issues are. You work with the community to fix them.

Graduates also credited UMed with sharpening their cultural competency and patient advocacy skills, such as Julia, an internal medicine physician and 2014 graduate:

It’s kind of hard for me to parse out what of that is from UMed and what’s just from my personal goals. But I do think I am able to relate to a lot of people more than a lot of my colleagues are, mostly because I try to figure out what’s actually important to patients and a lot of times it’s not things that we think it should be like your screening for a year, getting a flu shot, or screening for diabetes. (…) I think it has helped me to pay attention to what patients are telling me as opposed to just having an agenda coming into a visit.

Likewise, graduates credited the curriculum’s focus on structural inequities as supportive of building essential skills. They felt empowered knowing that individual efforts and systematic changes are equally important to the clinical context. Maria (CO 2014), who practices family medicine, remarked:

I feel like I do have a better sense of the barriers the patients can experience. Maybe a little bit more humility about knowing that I can’t change, we can’t fix everything, but at least finding a way to either help someone finding cheaper medications through a coupon, small things that you can do can mean a lot for patients, even if you’re not solving the entire problem.

Feli (CO 2013), a surgeon, shared how the curriculum empowered her to tackle systems-level changes:

I have a set of skills that have empowered me to try to improve our very complex healthcare system, even if it’s in a tiny way, so maybe I’ll just feel a little bit more empowered as opposed to saying that I have to accept this is the system (…). Because [in UMed] we had to explore cases, read books, understand the structural barriers to care, structural racism, like really explore it beyond the learning, the biochemistry of the medications to treat hypertension.

Although many felt that they gained practical and long-lasting skills from the UMed curriculum, this was not universally true. Ronny (CO 2020), an emergency medicine physician, stated,

I will be honest to say that I don’t think UMed truly changed the direction of my practice, or what I was gonna end up doing. I think for me the biggest take home was less about UMed in itself, but more about my community partner. I still interact and engage with the lead from my community partner. And I think that was on me more so than like the program.

With Ronny’s predisposition to work in urban underserved communities, the strongest and most long-lasting impact was the relationship he built with his community partner.

Fatima (CO 2020), a psychiatrist, similarly came to UMed with extensive community engagement experience prior to medical school: “[UMed] wasn’t really a skills-based experience to me (…) I had already worked with organizations in the community,” and she went on to identify opportunities for the curriculum to more intentionally teach competencies, “Like meeting facilitation, group facilitation, communication, theories around organizational structure and things like that.”

Despite differing perspectives on how the curriculum contributed to their development, most graduates could identify differences in how they practice medicine compared to their colleagues. When asked how their medical practice differs from colleagues who did not participate in a program like UMed, Alex (CO 2019), an internal medicine physician, attributed this difference to greater patience with patients experiencing low health literacy and a stronger commitment to advocacy:

I’ve noticed that my colleagues aren’t as great at reading through the lines, and certainly being an advocate for their patients when the patients can’t be an advocate for themselves. Having the patience with the patient population that has low health literacy, I think, is something that was kind of drilled in me as part of the UMed curriculum, and I know that a lot of my colleagues certainly don’t have that (…).

Furthermore, in Thomas’ case, a family medicine physician (CO 2012), he shared:

As a white male practicing in predominantly minority communities, I think I’m more comfortable and my patients are a lot more comfortable with me because of some of the skills and perspectives I’ve garnered over a significant amount of time. UMed was an important experience in molding my perspective about treating patients in my predominantly minority communities.

These reflections illustrate how UMed graduates apply a holistic, equity-focused approach to patient care, integrating cultural competency, structural awareness, and patient advocacy into their practice. Their experiences highlight the program’s lasting impact on reinforcing students’ values while shaping physicians attuned to structural competency and committed to addressing systemic barriers in healthcare through advocacy.

### Belonging and support as mechanisms for sustaining equity-oriented commitments

While the first two themes reflect direct influences on graduates’ clinical practice, participants described belonging and support as key mechanisms through which UMed shaped their careers and helped sustain equity-oriented commitments over time. Relationships formed through the cohort model reinforced shared values and helped participants remain committed to serving underserved communities, even as they pursued different specialties and career paths. As Julian (CO 2011), an internal medicine physician, reflected, “we all had a (…) passion for taking care of the underserved. We took different paths, but still have that core mission in place.”

During training, this sense of a shared purpose cultivated belonging and helped participants persist in medical school while remaining aligned with their professional goals, particularly in environments that did not consistently reinforce equity-oriented values. Graduates described UMed as providing both emotional support and a practical anchor for their career direction. Lucy (CO 2019), a pediatrician, explained that during the early years of medical school, which felt “really grueling and grinding,” opportunities through UMed to engage with patients and community-based work were “really important in trying to keep me driven towards my eventual goal of being a clinician.”

However, not all graduates experienced this same sense of belonging, or even safety. Demonstratively, Fatima remarked that she did not feel that she, or other students, could always be forthright about their thoughts or experiences due to a lack of grounding principles:

I imagine probably there are students who want to talk who aren't sure if they can [ ... ] because you feel like there aren't a set of grounding principles that people really agreed upon. And so, if I say something that makes someone else uncomfortable, like their uncomfortability is prioritized over someone else's very legitimate safety. [ ... ] We didn't have explicit conversations about those types of things early on to set the kind of groundwork for that.

Despite this experience, when asked whether she would participate in UMed again, she indicated that she would. Nonetheless, this lack of grounding principles during Fatima's academic experience with UMed signals that further community and trust-building were warranted.

Overall, most participants linked their relationships with UMed classmates to their long-term professional trajectories. Graduates described maintaining connections with UMed peers and mentors that influenced where they chose to work, the types of roles they pursued, and the communities they continued to engage. Ashley (CO 2011), a family medicine physician, explained that she chose to “work in places where I’m most needed,” drawing on prior experiences and relationships in making those decisions. Others described how UMed relationships directly shaped their career paths. Nick (CO 2017), an OB-GYN, who remains connected to peers across cohorts, shared that one UMed colleague “(…) was the person who introduced me to the field that I’m now in and we are very close.” Ray (CO 2013), an internal medicine physician, also reflected that he has witnessed many members of his cohort go on to leadership, nonprofit, and community-based roles, “doing really great stuff…giving back… and have already made an impact in medicine.”

Long-term impact was also evident in sustained professional and community collaborations with UMed community partner sites. Kevin (CO 2015), an OB-GYN, described reconnecting with a former UMed site years after graduation:

We did multi-site research on Asian-American women. (…) it was really important because her patient population has a large volume of Asian-Americans with limited English proficiency that my fellowship doesn’t necessarily see so it helps me to really diversify my sample, my scholarly pursuit.

Similarly, Alex described how relationships formed through his UMed community site continued well into professional practice:

I still work with [community site] to this day, and those are connections that I built that were very strong. (…) We brainstorm ideas and how to get things done and what to work on at [institution]. And they’re people that I still rub shoulders with when we’re teaching medical students.

Beyond shaping career trajectories, these relationships remained an important source of support as graduates navigated demanding clinical work, particularly in high-need settings. Ashley described how UMed peers continued to support one another during the pandemic by “shoring each other up and exploring burnout, and what it means to be in medicine,” adding that UMed was valuable for “knitting us together when we were young to be able to support each other longitudinally.” In this way, belonging functioned not only as a support during training, but an ongoing professional resource. Beyond professional life alone, over half (68.5%) of participants reported that they remain in contact with their UMed peers, with varying degrees of intimacy and regularity.

Graduates also described mentorship within UMed as critical to this process, providing role models who shaped their aspirations and modeled equity-oriented practice over time. Alex reflected on UMed leadership as “leaders in medicine that I could one day aspire to be similar to and I wouldn’t have gotten that elsewhere outside of UMed.” Simona similarly expressed that mentorship within the program “allowed me to be a better clinician and surgeon here for many, many years into practice at this point.” For some, the cumulative impact of these relationships was profound. Carmen (CO 2016), a pediatrician, reflected, “I feel like I get emotional because it’s why I’m the doctor that I am.” These findings highlight how belonging and mentorship influenced graduates’ practice in indirect but meaningful ways by shaping career decisions, sustaining community-engaged work, and providing ongoing support for equity-oriented practice.

### Participant recommendations for program improvement

While most participants expressed satisfaction with the program, a handful offered constructive feedback for improvement. Recommendations included: (1) establishing an alumni network to strengthen long-term support; (2) providing more opportunities to apply structural competencies into clinical practice; (3) establishing more intentional community building early on within cohorts; (4) ongoing, critical examination of the impact of SL projects on community, and (5) providing more structured mentorship opportunities. Subsequent iterations of the program have since incorporated many of these suggestions. For example, UMed has built opportunities for alumni networking and mentorship of current students, incorporated policy and advocacy work and reflective practices, and developed a UMed orientation for incoming students to connect and set group norms pre-matriculation. Furthermore, recent research efforts focus on capturing the experiences of community members served by UMed students. UMed continuously elicits feedback from students and stakeholders to guide program improvement and direction. While not a dominant theme, these recommendations invite reflection and opportunities for UMed and other SL programs.

## Discussion

This study explored the long-term impact of a longitudinal service-learning program on physicians’ professional development and clinical practice, particularly as it relates to reinforcing health equity training. Prior research has established that medical training is frequently associated with declines in empathy, increased cynicism, and a diminished attention to social and structural determinants of health– changes attributed, in large part, to the hidden curriculum, in which implicit institutional norms shape trainees’ attitudes and behaviors [11–15, 25]. Despite growing recognition of this problem, there remains a gap in the literature regarding whether longitudinal, community-engaged educational structures can sustain equity commitments across a physician’s career. Our findings suggest that longitudinal service-learning may have durable effects on physicians’ professional identities, approaches to patient care, and ongoing commitment to health equity. Participants consistently described UMed as reinforcing their equity-oriented values, helping translate structural competency into clinical practice, and providing a professional community that sustained their commitments to health equity and underserved care. Importantly, these findings remained consistent across cohorts.

A key contribution of this study is the finding that participants’ pre-existing interests in underserved care were not static. Although many graduates entered UMed with a strong commitment to health equity, as it was a prerequisite for program admission, they described the importance of continued reinforcement of these values throughout medical school. The longitudinal community rotation (LCR) provided a consistent point of connection to the patient populations they aimed to serve, helping to anchor their sense of purpose and counterbalance competing pressures within the broader training environment. This finding suggests that reinforcing structures may be necessary even for highly motivated trainees.

In addition to sustaining commitment to health equity, UMed facilitated the translation of structural competency into clinical practice. Graduates described applying an expanded understanding of social determinants of health, engaging in patient advocacy, and adopting a more holistic approach to care across specialties, often in contrast to their colleagues who did not participate in a similar program. These findings extend prior work on service-learning by demonstrating that longitudinal, experiential models may have durable effects on how physicians conceptualize and enact their roles in practice. [21–22, 26] Where earlier studies have largely documented short-term attitudinal outcomes, our findings imply these effects persist into clinical practice, representing a meaningful addition to the evidence base for community-engaged medical education.

The small cohort-based structure and emphasis on mentorship also emerged as critical mechanisms for sustaining equity-oriented commitments over time. Participants described their UMed community as providing a sense of belonging, validation, and support, particularly when their values were not consistently reflected in the broader medical practice environment. While this support facilitated persistence during training, participants more notably described how these relationships extended beyond medical school and continued to shape their professional trajectories and clinical practice. Graduates described maintaining relationships with peers and mentors that influenced where they chose to work, the roles they pursued, and their ongoing engagement in underserved communities. These findings suggest that belonging operated not only as a feature of the learning environment, but as a form of longitudinal professional infrastructure that supported the persistence of equity-oriented values and behaviors in practice. These findings further align with existing literature on the importance of social support and community in professional identity formation, but extend prior work by demonstrating how relational structures may also shape long-term career decisions and sustain equity-oriented commitments [27–29]. 

These findings indicate that longitudinal SL programs may serve a dual function in medical education: cultivating equity-oriented skills and perspectives, while also sustaining these commitments in the face of competing institutional influences. This has important implications for curriculum design, policy, and practice. Medical education may benefit not only from incorporating content related to health disparities and structural competency, but from creating longitudinal, community-engaged experiences and supportive learning environments that reinforce these values over time. From a policy perspective, institutional leaders and accreditation bodies might consider how structural support for SL programming (protected time, dedicated funding, formal recognition within curricula) can be embedded into standards for undergraduate medical education. The findings underscore the importance of mentorship and community, not just for enrichment, but as essential scaffolding for physicians. As patient populations continue to grow and diversify, the need for competent physicians trained in structural competency will likewise grow [11]. As such, key recommendations based on this study’s findings are: (1) offer sustained SL engagement opportunities in underserved settings; (2) provide small, cohort-based programs to support students in navigating training and reinforcing their professional goals; and (3) integrate structural competency training to address systemic barriers to health.

### Limitations

Several features of this study’s design create important considerations for how these findings should be interpreted and applied. Participants self-selected into both the UMed program and this study, which may reflect a sample predominantly composed of graduates with positive program experience. This is further reflected by the fact that only a few respondents had strongly critical feedback about their program experience. This context may have influenced the nature of the results in that, rather than a representative account of all possible outcomes of SL, our findings illuminate the mechanisms and experiences of a motivated, equity-committed cohort. Notably, this framing strengthens one of our core claims that even highly motivated individuals benefit from sustained and ongoing support and reinforcement. Even so, it must not be overlooked that opportunities for bias were introduced. We acknowledge that some interview questions were unintentionally framed in such a way that assumed particular outcomes of participant’s experiences with the program, potentially influencing how participants responded. Several participants themselves commented that it was difficult to disentangle the influence of UMed from their own intrinsic values and professional goals. Finally, the retrospective design and the passage of time since graduation may have introduced the possibility of recall bias. Participants reflecting on earlier iterations of the curriculum may not accurately represent how UMed currently operates, and readers should apply these findings to current program iterations with appropriate caution. Finally, the absence of a comparison group that didn’t participate in UMed limits our ability to attribute outcomes solely to UMed. Future research could build on these findings through comparative or longitudinal designs.

## Conclusions

This study highlights the lasting impact of a longitudinal medical school curriculum focused on health disparities and experiential learning on graduates’ clinical practice and commitment to underserved communities. The program’s emphasis on community, longitudinal engagement, mentorship, and structural competency uniquely prepared graduates to navigate complex healthcare environments with a holistic, patient-centered approach. These findings suggest that longitudinal service-learning programs may be critical not only for cultivating, but for sustaining commitments to health equity, particularly within training environments where such commitments may otherwise diminish over time. Future research into the impact that SL programs have on the communities being served, and whether these outcomes replicate across institutional contexts, is warranted.

## Supplementary Information

Supplementary Material 1.

## Data Availability

The datasets generated and/or analyzed during the current study are not publicly available due to concerns regarding participant confidentiality and the potentially identifiable nature of qualitative interview data but are available from the corresponding author on reasonable request.
