# Supplementary material for: “It’s why I’m the doctor that I am”: a qualitative study of the long-term outcomes of a longitudinal service-learning medical education program
Source: BMC Med Educ. 2026 Aug 10;26:1452. doi: 10.1186/s12909-026-10117-w (PMC13560443; doi:10.1186/s12909-026-10117-w)
Supplement: Supplementary file 1 [file 12909_2026_10117_MOESM1_ESM.docx]

**Appendix A- Semi-Structured Interview Guide**

1. What has your career path been like since medical school?
   a. Probe: Are you seeing patients, teaching, conducting research?
   b. Probe: If you’re seeing patients, how would you describe your patient population?
   c. Probe: What about other pursuits outside of medicine? (Leadership opportunities, continuing education, etc.)
2. Are there currently specific populations or healthcare topics that you are particularly passionate about? What do you believe influenced you in pursuing those interest?
3. Can you share a little bit about why you chose to participate in the UMed Program?
4. What were the take home messages for you?
5. What were the most meaningful experiences for you?
6. What was it like to move through medical school with your UMed cohort?
   a. Probe: What do you recall about your cohort experience?
7. Do you stay in touch with other UMed graduates? How do you support each other?
8. Can you share a bit about your community project and how it impacted your experience in medical school.
9. In what ways do you feel you made an impact on the individuals that you served through UMed?
10. Overall, what was the value of UMed towards your medical school experience?
11. Now we’ll transition to reflect on how UMed has impacted your career to date. To what extent do you apply UMed training and experiences to your current practice?
    a. Probe: Are there any specific tools or trainings that are currently relevant to your practice? How so?
    b. If not, why do you think that is?
12. How did your UMed experience influence your specialty choice, if at all? Other choices post-graduation?
13. What are some of your long-term career goals, and do you believe UMed influenced this? How so?
14. How do you feel UMed has influenced how you practice compared to your colleagues?
15. Based on your current expertise and experiences, what do you think are the most important topics for UMed students to be learning about when practicing medicine in disinvested communities?
    a. Probe: If you could go back, knowing what you know now, what would you have wanted to learn?
16. What do you wish you would have learned to be better equipped as a practitioner?
17. What would you add or change about the program?
18. If you could go back to medical school would you repeat the program? Why or why not?
19. Should UMed be required of all medical students? Why or why not?
20. And to conclude, is there anything else that you would like to say about the impact that your UMed experience has had on your personal and professional growth?
